# Artificial intelligence and immersive digital technologies in periodontal education: a systematic review

**DOI:** 10.3389/froh.2026.1741033

**Published:** 2026-04-09

**Authors:** Yiping Wei, Ying Li, Wenjie Hu, Jun Kang, Ziyao Han, Min Zhen, Cui Wang

**Affiliations:** Department of Periodontology, National Center for Stomatology, National Clinical Research Center for Oral Diseases, National Engineering Research Center of Oral Biomaterials and Digital Medical Devices, Peking University School and Hospital of Stomatology, Beijing, China

**Keywords:** artificial intelligence, dental education, digital learning, immersive technologies, periodontics

## Abstract

**Objectives:**

The purpose of the systematic review was to evaluate the application and efficacy of artificial intelligence (AI) and immersive digital technologies in periodontal education.

**Methods:**

We conducted a comprehensive search of PubMed, Embase, Web of Science, and Cochrane Central Register of Controlled Trials up to July 2025, supplemented by manual searches. Risk of bias was assessed using the Cochrane Risk of Bias 2.0 tool for randomized controlled trials and the Joanna Briggs Institute checklists for quasi-experimental and analytical cross-sectional studies.

**Results:**

Fifteen studies encompassing 3062 dental trainees and practitioners were included. Immersive digital technologies, including haptics-based virtual reality (VR), 360°VR, and virtual patient simulations, improved procedural skills, learner engagement, and communication abilities, particularly when combined with traditional training. AI applications such as explainable AI, AI-enhanced imaging, and large language models (LLMs) showed mixed outcomes. AI-assisted diagnostic tools offered limited advantage over conventional methods and may introduce automation bias. LLMs displayed variable accuracy and reliability.

**Conclusions:**

Dental educators should use blended, sequenced immersive digital technologies to enhance procedural and communication skills. AI diagnostic tools require safeguards against automation bias. LLMs can assist with grading but are unreliable as test-takers. Future multi-center randomized controlled trials are needed to assess long-term effectiveness and cost-efficiency.

**Systematic Review Registration:**

https://www.crd.york.ac.uk/PROSPERO/view/CRD420251027251, PROSPERO CRD420251027251.

## Introduction

Periodontal diseases pose a significant global health burden, contributing to tooth loss and adversely affecting systemic health (1–4). Consequently, the growing demand for competent periodontal care underscores the critical importance of robust professional education, rigorous clinical training, and practical experience for dental students. As a clinically oriented discipline with strong practical applicability, periodontal education has traditionally relied on manikin-based simulation equipment and employed a structured four-step approach: theoretical instruction, demonstration, student practice, and instructor evaluation (5). While this model has proven effective, it has notable limitations. Insufficient opportunities for practical training remain a persistent challenge, constrained by time allocation, spatial capacity, and teaching resources. Consequently, students often report inadequate development of clinical skills, diminishing their confidence for independent clinical practice (6). Furthermore, traditional teaching methods are often less effective at cultivating non-technical skills such as communication, critical thinking, and teamwork.

The global trend toward the digital transformation of education offers new opportunities to address these challenges. There have been a great number of technological advances over the last decade, particularly in the fields of artificial intelligence (AI) and immersive digital technologies (7, 8). AI technologies such as machine learning, deep learning, natural language processing, and large language models (LLMs) enable adaptive learning, automated evaluation, and timely feedback (9, 10). Immersive modalities such as virtual reality (VR), augmented reality, and virtual patient platforms can provide repeatable, highly interactive and realistic simulations, thereby enhancing both practical skills and clinical decision-making (11). Within medical and dental education, these technologies have demonstrated potential to improve knowledge retention, procedural performance, diagnostic accuracy, and learner engagement (12–15).

In the specific context of periodontology, AI has been explored for applications such as radiographic interpretation, predicting prognosis, and automated grading (16–18) Alongside, immersive tools have been applied to train essential procedures (e.g., periodontal probing, scaling, and root planing) and to develop communication skills through simulated patient interactions (19–21). Despite these advances, evidence on their effectiveness, optimal integration into curricula, and potential limitations in periodontal education remains fragmented. Previous reviews have primarily summarized AI applications in periodontal clinical practice (22–24). However, to our knowledge, no systematic review has comprehensively evaluated their role in periodontal education. While the scoping review by Saghiri et al. (15) examined AI and immersive digital technologies within broader dental education, few have focused specifically on periodontal training for both learners and educators. Periodontal education involves unique challenges, including the development of fine motor skills for procedural tasks, the interpretation of periodontal imaging and clinical indices, and long-term treatment planning. As such, this review focuses specifically on outcomes pertinent to periodontal education, mapping technologies to critical curriculum decision points, and offering practical, domain-specific guidance. This approach addresses a crucial evidence gap, as educational contexts have distinct requirements and challenges that differ from clinical applications.

This systematic review aims to synthesize current evidence on the application of AI and immersive digital technologies in periodontal education. Specifically, it evaluates the types of technologies employed, their educational purposes and measured outcomes, methodological quality of the studies, and the challenges reported. The goal is to provide evidence-based insights to guide curriculum development and identify priorities for future research and implementation.

## Methods

### Protocol and registration

The present study was developed following the Preferred Reporting Items for Systematic Reviews and Meta-Analyses (PRISMA) checklist and the protocol was registered with the National Institute for Health and Care Research (NIHR) under the PROSPERO ID CRD420251027251. During the literature screening process, immersive digital technologies, particularly VR, emerged as a closely related yet distinct domain within periodontal education. Accordingly, the review title and scope were refined to reflect the thematic synthesis of the included studies. A PRISMA 2020 checklist is provided in the Supplementary Material, detailing the specific location of each reporting item within the manuscript.

### Focused research questions

The focused research question of this systematic review was formulated according to the PICOS framework (Population, Intervention, Comparison, Outcome, and Study Design): Among learners and educators involved in periodontal education (P), how do artificial intelligence (AI) and immersive digital technologies (I), compared with traditional teaching methods or no digital assistance (C), influence educational outcomes such as knowledge acquisition, clinical skill development, diagnostic performance, and learner engagement (O) in original research studies (S)?

### Eligibility criteria

The inclusion criteria of this systematic review were organized by the modified PICOS framework: (P) Participants. Students, trainees, residents, practitioners and educators involved in periodontal education and training. (I) Intervention. Immersive digital technologies, including VR, haptic simulators, virtual patients, and augmented reality, or AI technologies, such as machine learning, deep learning, and large language models (LLMs), applied to periodontal education. (C) Comparison. Traditional teaching methods, such as lectures, textbooks and manual clinical training, were used as the comparator. However, it was not a mandatory item to include a study in this review. (O) Outcome measures. Analysis of the effectiveness of AI and immersive digital technologies in improving knowledge, practical skills, decision-making, critical thinking, and learner engagement. Additional measures included the accuracy and reliability of AI tools, user satisfaction, and the perceived value of immersive learning environments. (S) Studies. Any original studies, including randomized controlled trials (RCTs), nonrandomized interventional studies, and observational studies, published in English were included.

The exclusion criteria were as follows: (1) Review articles, conference abstracts, editorials, and commentaries; (2) Studies not directly focused on periodontal education; (3) Articles not available in full text.

### Search strategy

A comprehensive literature search was conducted through electronic databases PubMed, Embase, Web of Science, and Cochrane Central Register of Controlled Trials using an ad-hoc search string that was adapted to each database: (“artificial intelligence” OR “AI” OR “machine learning” OR “deep learning” OR “natural language processing” OR “neural network” OR “virtual reality” OR “augmented reality” OR “mixed reality” OR “immersive technology” OR “intelligent tutoring system” OR “AI-based learning” OR “computer-assisted instruction” OR “chatbot” OR “educational technology”) AND (“dental education” OR “dental curriculum” OR “dental student” OR “periodontal education” OR “periodontology education” OR “teaching periodontics” OR “periodontics training”). The detailed search strategy for each database are available in the Supplementary Table S1.

Databases were searched for studies published between January 1, 2000, and June 30, 2025, with a final manual update conducted on July 30, 2025, to capture the most recent studies. Furthermore, a manual search of the reference lists of the included studies, journals of interest, and relevant reviews was performed. All records were imported into EndNote (version X9.3.2, Clarivate Analytics), which automatically removed the duplicates. Resulting list was further manually screened for extra duplicates. Studies identified through manual searches were recorded in the PRISMA flow diagram under “other sources”.

### Study selection and data extraction

Studies titles and abstracts were screened independently by two review authors (YW and YL) to identify candidate studies for the eligibility criteria. Irrelevant records were excluded, and conflicts were resolved with discussion. Afterwards full text articles were evaluated to include those who reach all the inclusion criteria to the final review. The level of agreement between the 2 reviewers was calculated by using kappa statistics. Any disagreement was fixed through discussion until consensus was reached or by consulting a third author (WH). The following data were extracted: study details (author, year, and country), study design, participant, educational scenario, assessed outcomes and main findings.

### Risk of bias assessment

The risk of bias for each included study was independently assessed by two reviewers (YW and YL). The Cochrane Risk of Bias Tool 2.0 (RoB 2) tool was used to assess the bias of RCTs (25). For nonrandomized experimental studies, the Joanna Briggs Institute (JBI) Critical Appraisal Checklist for Quasi-Experimental Studies was applied (26). The risk of bias of cross-sectional studies was assessed using the corresponding JBI Critical Appraisal Checklist for Analytical Cross-sectional Studies (27). Both checklists require reviewers to answer a series of questions by selecting “Yes”, “No”, “Unclear” and “Not applicable”. Disagreements between reviewers were resolved through discussion to reach a consensus. If an agreement could not be reached, a third reviewer (WH) was consulted for the final decision. Due to substantial heterogeneity in study designs, interventions, outcome measures, and educational settings, a quantitative meta-analysis was not performed. As no meta-analysis was conducted, items related to quantitative synthesis and publication bias assessment in the PRISMA checklist were considered not applicable.

## Results

### Study selection

**Figure 1:**
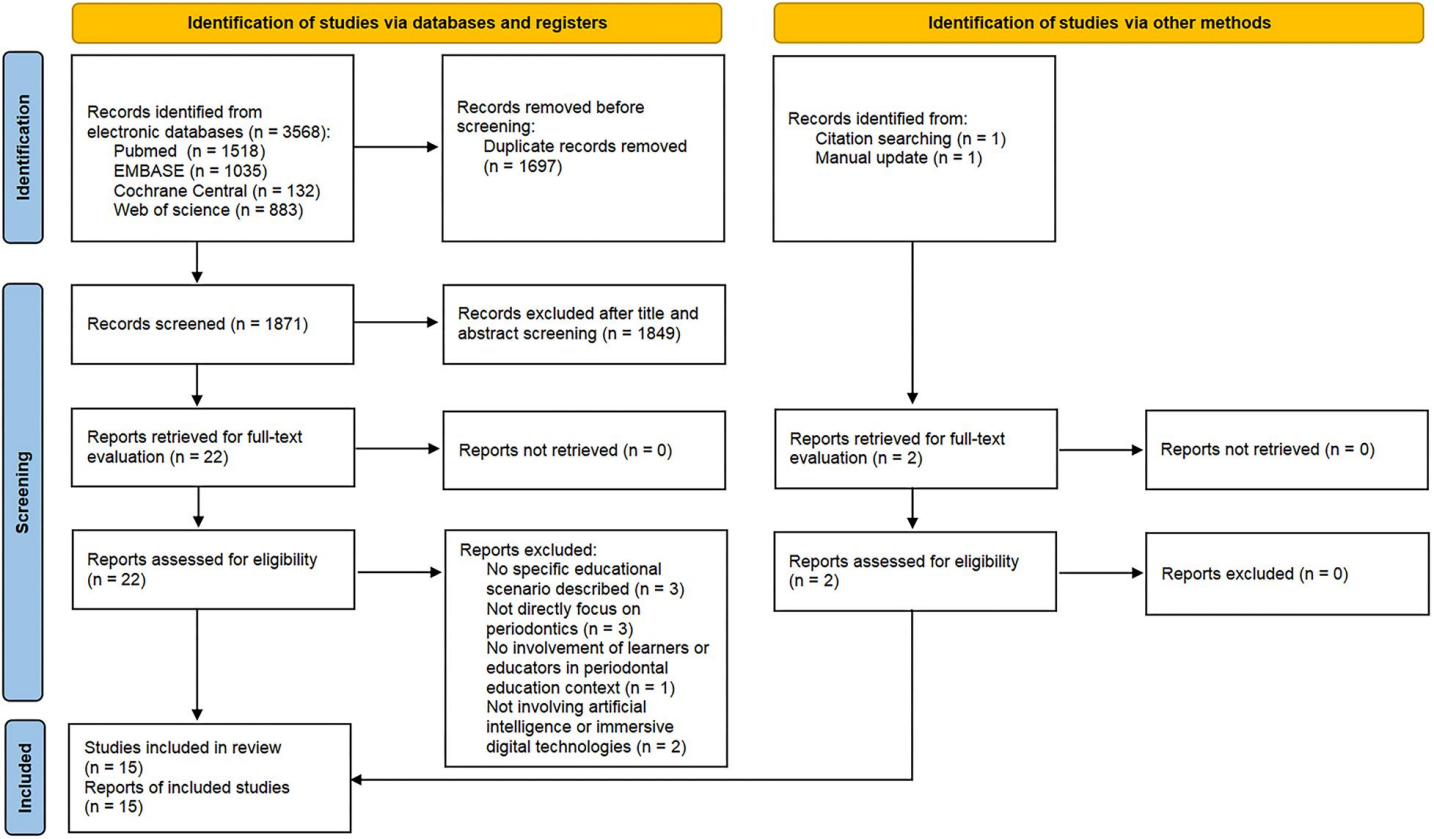
Flowchart of the study selection process.

The initial search identified 3568 references. After the removal of duplicates, the total number of entries was 1,871. A total of 1,849 articles were excluded after reviewing titles and abstracts. Out of 22 articles retrieved for full text assessment, 9 articles were excluded with reasons and listed in Supplementary Table S2. Kappa score for inter-examiner agreement for title and abstract review was 0.88. While for the full-text review phase, the value was 0.96 for the two assessors, demonstrating an excellent agreement. A manual search of reference lists and an updated search identified 2 additional articles. Eventually, 15 studies were considered suitable for inclusion in the systematic review. The flowchart of the aforementioned selection process is illustrated in Figure 1.

### Study characteristics

**Figure 2:**
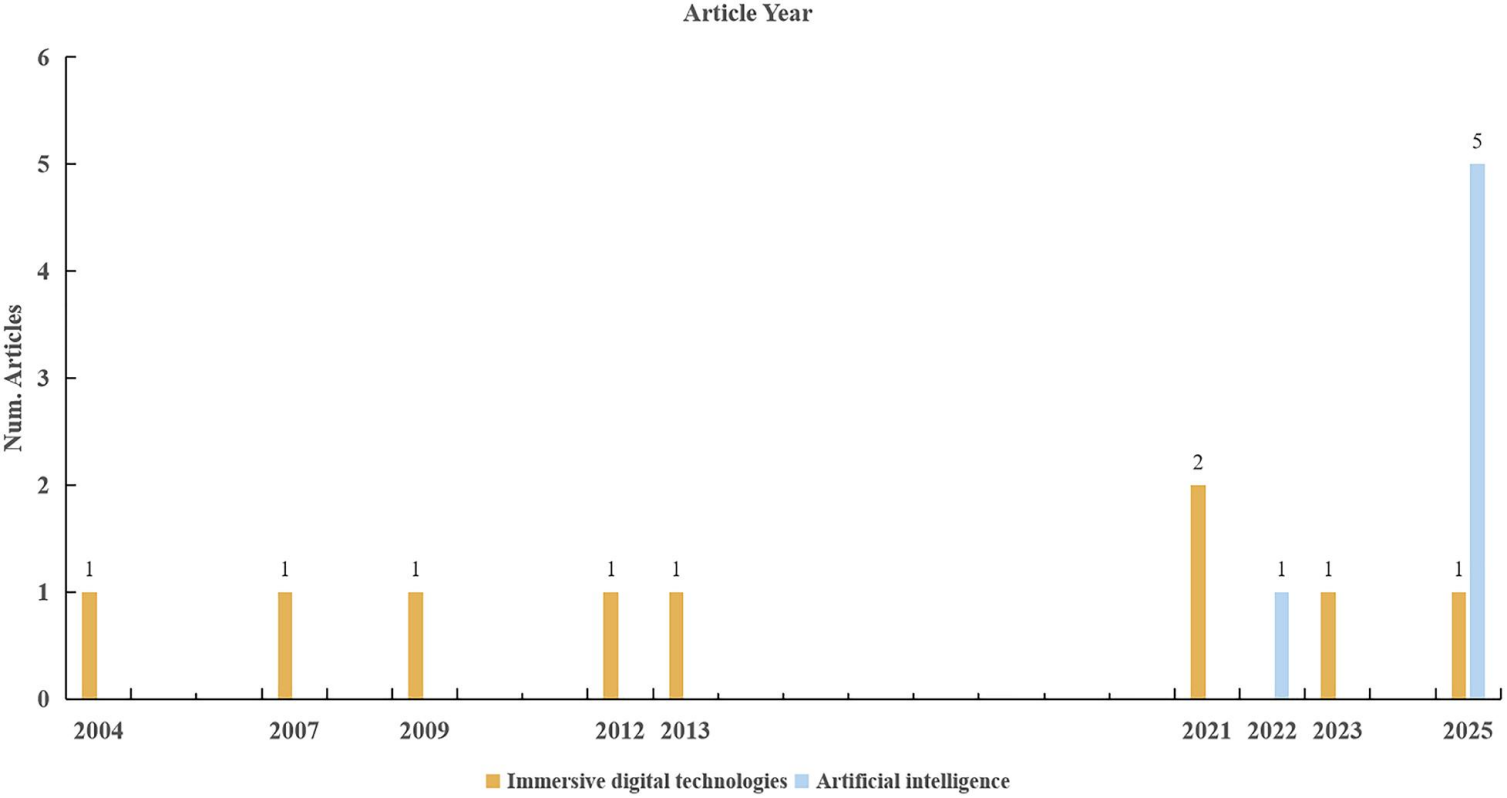
Distribution of included studies by publication year and category.

**Figure 3:**
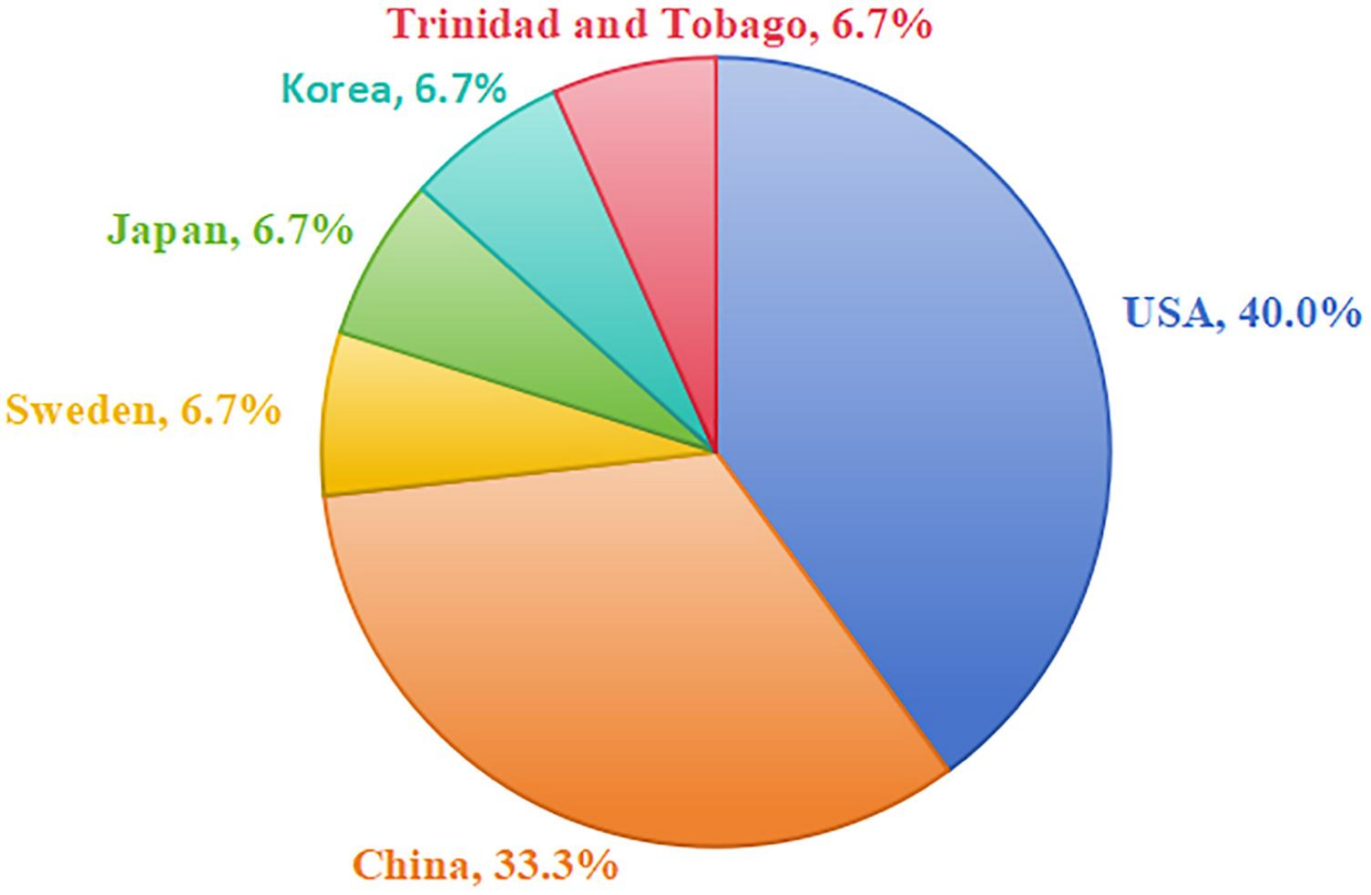
Distribution of included studies by country.

Table 1 presents a detailed overview of the included studies. Additionally, Supplementary Table S3 summarizes the technical specifications of the employed technologies, including the specific types of AI, platforms or models used, and their respective software versions. The included studies were published between 2004 and 2025, with authors representing 6 countries (Figure 2). The majority of the studies were conducted in the USA (*n* = 6) and in China (*n* = 5), with others from Sweden, Japan, Korea, and Trinidad and Tobago (Figure 3). Among these, 7 were RCTs (19, 21, 28–32), 2 were non-randomized interventional studies (20, 33), and 6 were cross-sectional studies (2 descriptive, 4 comparative) (34–39). The studies involved a total of 3,062 undergraduate dental students, postgraduate trainees, and faculty members. Among the included studies, 9 focused on the application of immersive digital technologies in periodontal education, primarily involving virtual patients, haptics-based VR, and 360° VR (19–21, 28, 29, 33–36). The remaining 6 studies explored the use of AI in periodontal education, covering explainable AI, AI-enhanced imaging, and LLMs (30–32, 37–39). The included studies addressed four primary domains of periodontal education: (1) procedural skill training, including techniques such as periodontal probing, scaling and root planing; (2) radiographic interpretation for periodontal diagnosis; (3) knowledge assessment; and (4) communication skills development. Both objective measures (such as test scores and performance metrics) and subjective measures (such as questionnaires and perception surveys) were reported.

**Table 1:** Characteristics of the included studies.

Study	Domain	Study design	Participant	Educational scenario	Assessed outcomes	Main findings
Schittek Janda et al. (19) (Sweden)	Virtual Patient	Randomized controlled trial	39 dental students in their second semester from the Centre for Oral Health Sciences, Malmö University.	The experimental group (16 students) worked with the virtual patient for 1 week prior to their first patient contact whilst the control group was first allowed to use the virtual patient after their first patient contact.	Use of time; Analysis of questions asked: number, quality, order; Professional behaviour: language precision, order of questions (logical sequence), empathy.	Students who trained with the virtual patient asked more critical and relevant questions, spent more time engaging with the patient before using notes, and demonstrated higher empathy and professional performance compared to controls.
Steinberg et al. (34) (USA)	Haptics-based virtual reality	Descriptive cross-sectional study (content validity study)	30 experienced clinical dental and dental hygiene faculty from the College of Dentistry, University of Illinois at Chicago.	Using the PerioSim© for periodontal probing simulation, including visual and tactile evaluation using a VR periodontal probe and explorer.	Realism of anatomical images and haptic sensations; Usefulness of instructional layout; Perceived usefulness for teaching and assessment.	The faculty rated the visual and tactile realism for teeth and instruments as high, but low for the gingiva. Overall, the simulator was perceived as a valuable adjunct for teaching and potentially effective for assessing basic periodontal skills.
Luciano et al. (35) (USA)	Haptics-based virtual reality	Descriptive cross-sectional study	18 faculty members and 5 students from the College of Dentistry, University of Illinois at Chicago.	Using the haptic simulator for 10 min to perform a simulated scaling and root planing procedure.	Participants rated the simulator's usability, perceived usefulness for teaching and course review and perceived realism of the haptic feedback.	The vast majority of participants found the simulator easy to use and adapted to it quickly. They rated it highly as a useful adjunct to traditional teaching and for future course review.
Wang et al. (36) (China)	Haptics-based virtual reality	Comparative cross-sectional Study (construct validity study)	10 expert dentists (>6 years experience) and 19 postgraduate students (1–2 years experience) from Peking University School and Hospital of Stomatology	Performing three simulated periodontal procedures on the iDental simulator: periodontal pocket probing, calculus detection and removal.	Qualitative: user ratings on the realism of visual/haptic feedback and ease of use. Quantitative: assessed construct validity by comparing expert vs. novice performance metrics (e.g., probing depth, force, calculus removal).	The simulator demonstrated construct validity for pocket probing by differentiating experts from novices, but not for the simpler calculus tasks. Users found the calculus simulation realistic but noted that probing feedback and other visual/haptic elements required improvement.
Yamaguchi et al. (20) (Japan)	Haptics-based virtual reality	Nonrandomized interventional study (pre-post test design)	26 fourth year dental students from Osaka University School of Dentistry.	Performing periodontal pocket probing on a haptic VR simulator over three repetitive training sessions.	Periodontal Pocket Probing score was based on the measured probing force, converted to a 1–5 score.	Scores progressively improved with each session, demonstrating the haptic VR simulator's effectiveness for short-term teaching of the periodontal pocket probing task.
Chehabeddine et al. (33) (USA)	Haptics-based virtual reality	Nonrandomized interventional study	32 faculty members from New York University College of Dentistry.	Using the Haptodont system, three groups of participants performed probing on healthy and periodontitis models. The groups were: Control (haptics only), Haptics plus Finger Support, and Haptics plus Finger Support and Vibrotactile Feedback.	Qualitative: post-experiment questionnaire on realism, usefulness, and suggestions for improvement. Quantitative: probing depth error (accuracy) and probing force.	Expert performance on the simulator met clinical standards for accuracy and force. The finger support feature significantly improved probing accuracy in difficult areas, whereas the vibrotactile feedback was distracting and worsened performance in difficult tasks.
Zhang et al. (28) (China)	Haptics-based virtual reality	Randomized controlled trial	60 s and third-year undergraduate students from the stomatology department in Lanzhou University,	Participants performed supragingival scaling and were randomized into four groups: jaw model only, VR (UniDental system) only, VR then jaw model, and jaw model then VR.	Theoretical: pre- and post-training knowledge scores. Practical: blinded scoring of supragingival scaling technique and plaque reduction. Questionnaire: user satisfaction scores.	Combined VR and jaw model training was significantly more effective than using either method alone for improving both theoretical and practical skills. The training sequence was critical, with the group training on the jaw model first and then VR achieving the best practical performance.
Tak et al. (29) (Korea)	360° Virtual reality	Randomized controlled trial	30 participants with no prior instrument training experience from Eulji University.	Participants were trained in hand scaling and randomly divided into three groups (*n* = 10): a control group using paper handouts, a group using 2D video, and a group using 360° VR video.	The primary outcome was the change in practical skill scores, measured at multiple stages (initial, mid-term, final).	Both 2D video and 360° VR were significantly more effective for skill acquisition than traditional paper handouts. 360° VR showed higher learning efficiency than regular 2D video,
Cheng et al. (21) (China)	Haptics-based virtual reality	Randomized controlled trial	90 undergraduate dental students from Guangzhou Medical University.	Participants learned to perform scaling and root planing techniques and were randomly divided into three groups (*n* = 30 per group): VR group, Model group (control), Combined group (VR plus Model).	Practical operation scores: total score and scores for specific skills including fulcrum, instrument angle, and force/motion application. Questionnaire scores: student satisfaction and perception of learning.	The combined teaching model yielded the best overall performance. It leveraged the traditional model's superiority for teaching “fulcrum” and VR's superiority for teaching “instrument angle”. The study concludes that this combined approach, utilizing the distinct advantages of each method, is the optimal strategy.
Glick et al. (30) (USA)	Explainable artificial intelligence	Randomized controlled trial	22 third- and 19 fourth-year dental students from the University of Texas School of Dentistry.	Participants were randomly assigned to one of two groups to identify furcation involvement on radiographs: a control group (*n* = 21) without AI assistance and a test group (*n* = 20) with AI-labeled radiographs.	Performance: diagnostic accuracy. Efficiency: time taken to complete task. Confidence: self-reported levels before and after the task. Perception: belief in AI's usefulness for decision-making.	AI assistance did not improve novice clinicians’ performance, efficiency, or confidence. Critically, it led to over-reliance, as the AI-assisted group was significantly more likely to agree with incorrect AI suggestions. Nevertheless, both groups believed AI has the potential to be a useful clinical tool.
Boehm et al. (31) (USA)	Artificial intelligence-enhanced imaging	2 × 2 factorial randomized controlled trial	13 first-year and 12 fourth-year predoctoral dental students from Western University of Health Sciences, College of Dental Medicine.	Participants, split by experience level (novice vs. experienced), were assigned to use either an AI tool (Overjet) or a conventional tool (MIPACS) to identify periodontal bone loss on 30 two-dimensional radiographs.	The change in diagnostic accuracy from a pretest to a posttest after a brief training intervention with either AI-enhanced or conventional imaging software.	While both novice and experienced students significantly improved their diagnostic accuracy post-training, the AI-enhanced tool offered no statistical advantage over the conventional one. Novices, however, demonstrated a significantly greater accuracy gain than experienced students.
Li et al. (37) (China)	Large language models	Comparative cross-sectional study	114 fourth-year dental students from West China School of Stomatology, Sichuan University, ChatGPT-3.5, and ChatGPT-4.	Answering a 25-item multiple-choice questionnaire on periodontal surgery; Answering an open-ended question simulating a clinical scenario.	Multiple-choice question accuracy; Response time (for ChatGPT); Evaluation of student answers to the open-ended question (ChatGPT-4 vs. teacher).	Students outperformed ChatGPT on multiple-choice questions, but ChatGPT-4 matched human teachers in evaluating open-ended answers. The study concludes that while not yet for exams, ChatGPT-4 is a promising assistant for curriculum and grading tasks.
Sabri et al. (38) (USA)	Large language models	Comparative cross-sectional study	Three LLMs (ChatGPT-4, ChatGPT-3.5, Gemini) were compared against a human control group of 2375 periodontal residents (postgraduate years 1, 2, and 3) who took the exams from 2020 to 2023.	Answering a corpus of 1312 multiple-choice questions from the American Academy of Periodontology annual in-service examination (2020–2023).	The primary outcome was the accuracy (percentage of correct answers) on the examination. The performance was also analyzed by exam section and on the most difficult questions (those with <50% resident accuracy).	With 79.57% accuracy, ChatGPT-4 significantly outperformed all human resident groups and the other AI models (Gemini, ChatGPT-3.5) in all exam years. Google Gemini performed better than ChatGPT-3.5 and surpassed first- and second-year residents, but not third-year residents. The study concludes that ChatGPT-4 is robustly accurate and reliable for this exam, while the other two AIs showed weaker performance.
Ramlogan et al. (39) (Trinidad and Tobago)	Large language models	Comparative cross–sectional study	22 final-year dental students from the University of the West Indies were compared against multiple LLMs, including various versions of ChatGPT-4, GPT-4o, Claude, Gemini, and DeepSeek.	The performance of various LLMs was compared to that of final-year dental students on a 20-item, short-answer periodontology exam. To assess consistency, ChatGPT-4's performance was also tracked longitudinally over 15 months.	Mean percentage score achieved on the written examination for both the student cohort and each of the LLMs.	Initially, ChatGPT-4 (78%) and GPT-4o (77%) significantly outperformed students (60%), as did other LLMs like Claude (87%). However, ChatGPT-4's performance proved inconsistent, dropping to the student level on a 6-month re-test. The study concludes that while LLMs show potential, they must be used with caution due to performance variability and potential inaccuracies.
Ma et al. (32) (China)	Large language models	Randomized controlled trial	126 undergraduate dental students (fourth-year) from the School of Stomatology, Binzhou Medical University.	Two groups of participants (*n* = 63 each) were randomly assigned to take a 90-item multiple-choice periodontology exam. The AI group took an exam generated by GPT-4, while the Human group took a traditional, human-designed exam.	Psychometric properties: exam reliability, content coverage, difficulty, and discrimination indices. Student performance: total examination scores. Student feedback: likert-scale ratings of perceived difficulty, knowledge coverage, and learning inspiration.	The AI-generated exam produced higher scores but failed to effectively discriminate between students. Students found it less inspiring and appropriately difficult than the human-designed exam. The study concludes AI lacks the discriminatory power for high-stakes assessments and recommends a hybrid “AI-human” approach.

AI, artificial intelligence; LLM, large language model; VR, virtual reality; 2D, two-dimensional.

### Main findings of included studies

#### Efficacy of immersive digital technologies on clinical skill acquisition

Multiple studies demonstrated that immersive digital technologies, particularly haptics-based VR and 360° VR, effectively enhanced students' periodontal practical skills. Repetitive training with haptic simulators led to significant improvements in task performance, such as better force control in periodontal probing (20). The integration of specific features like a haptic finger support mechanism, implemented in the Haptodont system, was shown to significantly improve probing accuracy, especially in difficult-to-access clinical scenarios (33). Furthermore, RCTs comparing teaching modalities consistently found that a blended approach combining traditional jaw models with VR training yielded the best overall performance in scaling and root planing skills (21, 28). One study specifically identified that the training sequence was important, with training on a jaw model first, followed by VR, yielding the optimal outcome (28).

### User perception and engagement with immersive digital technologies

Subjective feedback from participants regarding these immersive digital technologies was generally positive. Users frequently rated haptic simulators as realistic, easy to use, and a valuable adjunct to traditional teaching methods (34, 35). The use of a virtual patient (a custom web-based learning environment) was found to enhance students’ empathy and professional communication skill (19). Despite this positive reception, specific limitations were also identified. For example, the tactile realism of the gingiva in haptic models was perceived as low (34). Certain features like vibrotactile feedback were sometimes found to be distracting and detrimental to performance (33).

### Performance of AI in diagnostic training

Studies employing AI for diagnostic training have reported consistent outcomes. Notably, no statistically significant differences were observed in diagnostic accuracy between students utilizing AI-assisted tools and those in control groups (using conventional software or no assistance) for the identification of periodontal bone loss or furcation involvement (30, 31). Boehm et al. (31) utilized Overjet AI, a professional commercial dental software, comparing it against conventional MiPACS imaging, while Glick et al. (30) employed a custom-developed U-Net Convolutional Neural Network (CNN) serving as an explainable AI research model. A critical finding from the CNN study was that AI assistance did not improve novice clinicians’ confidence and introduced a risk of automation bias, where students showed a significant tendency to agree with an erroneous AI-generated answer (30).

### Application of LLMs in knowledge assessment

The performance of LLMs in knowledge assessment varied depending on the context and comparison group. One study found that a high-performing model, ChatGPT-4, could significantly outperform senior periodontal residents on standardized in-service examination (38). However, another study focusing on concepts of periodontal surgery presented a contrasting outcome, where dental students achieved a higher accuracy on multiple-choice questions (21.51/25) than both ChatGPT-4 (20/25) and ChatGPT-3.5 (14/25) (37). Interestingly, the same study noted that ChatGPT-4's evaluation of students’ open-ended answers was consistent with a human teacher's review, highlighting its potential as an assessment assistant rather than a test-taker. When the role of the LLM was shifted to test-creator, an RCT found that while the AI-generated exam provided broader content coverage, it had a significantly lower discrimination index, making it less effective at distinguishing between high- and low-performing students compared to a human-designed exam (32). Furthermore, students rated the AI-generated exam lower in terms of appropriate difficulty and learning inspiration. Concerns about reliability were also highlighted in a longitudinal study where ChatGPT-4's performance was found to be inconsistent, dropping significantly when re-tested months later (39).

### Risk of bias

**Figure 4:**
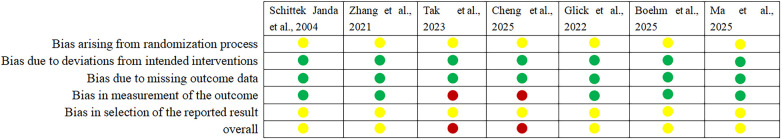
Risk of bias assessment for randomised controlled clinical trials using RoB 2.0 tools presented with low (green), some concerns (yellow) and high (red) risk of bias.

**Figure 5:**
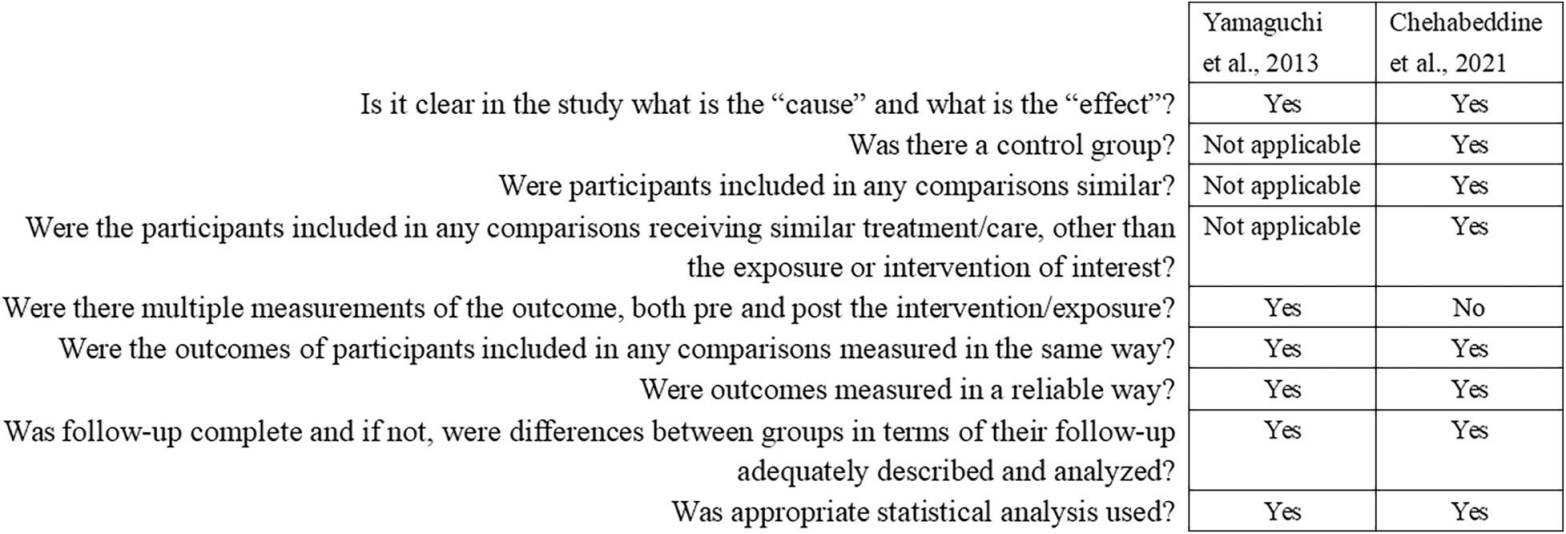
Risk of bias assessment based on joanna briggs institute critical appraisal checklist for quasi-experimental studies.

**Figure 6:**
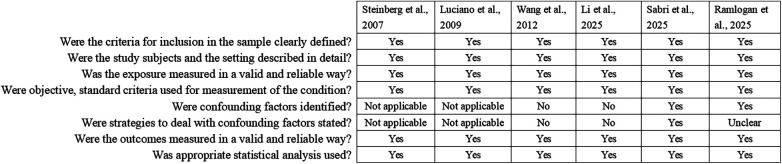
Risk of bias assessment based on joanna briggs institute critical appraisal checklist for analytical cross sectional studies.

Seven RCTs were assessed by the RoB2 tool and presented with some concerns to high risk (Figure 4). Across all included trials, the domain assessing the randomization process was rated as having some concerns, primarily due to insufficient details regarding randomization procedures and inadequate reporting of allocation concealment. Similarly, all studies were assessed as having some concerns for bias in the selection of the reported result, as none provided a pre-registered study protocol. Notably, two studies were judged to be at high risk of bias in the measurement of outcomes, owing to the lack of blinding in outcome assessment and the subjective nature of the employed outcome measures (21, 29). Other domains, including deviations from intended interventions and missing outcome data, were generally assessed as having low risk. Two nonrandomized interventional studies were assessed with the JBI Critical Appraisal Checklist (Figure 5). The first study adopted a pre-post test design without a control group; therefore, the three items related to the presence of a control group, similarity of participants in comparisons, and similarity of treatment other than the intervention were rated as “Not applicable” (20). The second study did not include a pre-intervention measurement, resulting in a “No” rating for the corresponding item (33). All other applicable domains were rated as “Yes”, indicating generally adequate methodological conduct, although design-related limitations should be considered when interpreting the findings. The included cross-sectional studies were assessed using the JBI Critical Appraisal Checklist for Analytical Cross-sectional Studies (Figure 6). For the two descriptive cross-sectional studies, items related to the identification of confounding factors and strategies to address them were rated as “Not applicable”, as these studies did not involve analytical comparisons (34, 35). For the remaining analytical cross-sectional studies, two did not identify or address confounding factors (36, 37), and one study provided unclear information regarding confounder identification (39). All studies clearly defined inclusion criteria, provided adequate detail on study subjects and settings, and measured both exposures and outcomes in a valid and reliable manner. Appropriate statistical analyses were reported in all studies.

## Discussion

This systematic review synthesizes evidence from 15 studies evaluating the application of AI and immersive digital technologies in periodontal education. Haptics-based VR, 360° VR, and virtual patient simulations can enhance procedural skills, communication, and learner engagement, particularly when combined with conventional training methods. AI-assisted diagnostic tools currently offer limited advantages over traditional approaches and may introduce automation bias without appropriate safeguards. LLMs demonstrate promise in assessment support and content generation but require careful oversight due to variable performance and reliability.

The publication timeline of the 15 included studies, spanning from 2004 to 2025, reflects a clear evolution in this area of research. Early work centered on feasibility studies for haptic simulators and virtual patient platforms. There is a marked acceleration in research output since 2020. The recent surge is dominated by investigations into more sophisticated AI applications, particularly diagnostic aids and the performance of LLMs. This shift indicates a maturation of the field from procedural skill simulation to the exploration of advanced cognitive tools for clinical reasoning and assessment.

Our results demonstrate that immersive digital technologies enhance procedural skills, in accordance with the broader body of evidence from medical and dental simulation research. Previous reviews have established that VR and haptic simulators provide safe, repeatable, and feedback-rich environments that can accelerate the learning curve for complex psychomotor tasks (40, 41). The consistent observation in this review that a blended approach, combining traditional jaw models with VR training, yields superior outcomes is particularly noteworthy (21, 28). This resonates with the principles of contemporary dental education, which emphasize that digital methods are most effective as enhancements to, rather than replacements for, traditional face to face and hands-on teaching (5). The finding that sequencing (jaw model training followed by VR) optimizes performance further suggests a need for strategic instructional design, where foundational tactile skills are developed on physical models before being refined in a more complex virtual environment. Nevertheless, the reported limitations, such as low tactile fidelity of the gingiva in haptic models, echo persistent challenges in simulator design and underscore the importance of ongoing technological refinement.

In the domain of diagnostic training, Glick et al. and Boehm et al. suggested a limited educational benefit of AI assistance (30, 31). However, it is important to note that compelling evidence exists from other clinical contexts where AI has been shown to significantly enhance diagnostic accuracy and efficiency (42, 43). A critical concern is the risk of automation bias, where students tend to over-rely on and agree with AI-generated suggestions even when they are incorrect (44, 45). This phenomenon has been repeatedly observed in other high-stakes decision-making settings, underscoring that the adoption of AI should not be viewed simply as replacing one diagnostic tool with another (46, 47). Instead, it calls for deliberate educational strategies aimed at strengthening learners’ capacity to critically appraise and, when necessary, challenge algorithmic output. In this context, AI may be best employed as a supplementary aid for broadening learners’ exposure to varied cases and highlighting features that warrant closer human review.

The performance of LLMs in knowledge assessment presents both opportunities and challenges, reflecting recent findings in dental and medical education (48, 49). The ability of models like ChatGPT-4 to outperform senior residents on standardized tests suggests their proficiency in knowledge recall and pattern recognition on well-defined tasks (38). However, their underperformance compared to dental students in complex, domain-specific reasoning (e.g., periodontal surgery concepts) reveals current limitations in deep conceptual understanding (37). Our findings support the emerging view of LLMs as powerful assessment assistants, capable of providing consistent evaluation of open-ended answers, rather than autonomous test-takers or test-creators. The lower discrimination index of AI-generated exams and concerns about their reliability over time further emphasize the need for human oversight and rigorous validation before their integration into high-stakes educational assessments.

Overall, the methodological quality of the included studies was moderate, with common weaknesses that warrant attention in future research. Most randomized controlled trials were rated as having “some concerns”, primarily due to the insufficient reporting of allocation concealment and the inadequate detail on randomization procedures. The absence of pre-registered protocols across all trials further limited transparency and increased the risk of selective reporting. The lack of blinding and reliance on subjective outcome measures occasionally elevated the risk of measurement bias. Although the non-randomized and cross-sectional studies generally demonstrated low risk in most domains, several failed to identify or address potential confounding factors.

The interpretation of our findings is limited by the exclusion of regional and non-English databases, which may have introduced potential selection and language bias. In addition, the small number of studies, considerable heterogeneity in designs, participants, and outcome measures, which precluded meta-analysis and reduced generalizability. Accessibility barriers should also be considered, as the high cost of VR equipment, infrastructure requirements, and varying levels of digital literacy may limit the adoption of these technologies in low-resource educational settings. Another limitation is that learner and educator perspectives were not consistently reported or analyzed separately in the included studies, which limited our ability to compare potential differences in experiences between these groups. Furthermore, the lack of long-term follow-up studies represents an important limitation. Short-term improvements may partly reflect novelty effects associated with new technologies. Longitudinal research is needed to evaluate sustained learning outcomes and skill retention. Future research should prioritize multi-center, adequately powered RCTs with standardized outcome measures and longer-term follow-up. Comparative effectiveness studies examining the optimal integration of AI and immersive digital technologies into existing curricula are warranted, alongside investigations into cost-effectiveness and faculty workload implications. For LLMs, continuous performance monitoring, domain-specific fine-tuning, and ethical governance frameworks will be essential to ensure safe and effective educational deployment.

## Conclusions

The existing evidence supports the AI and immersive digital technologies as adjunctive tools within periodontal education. Their optimal use requires strategic instructional design, faculty training, and continuous evaluation. Further multi-center, methodologically rigorous studies are essential to establish long-term educational outcomes, cost-effectiveness, and safe implementation pathways.

## Data Availability

The raw data supporting the conclusions of this article will be made available by the authors, without undue reservation.
