# Supplementary material for: Artificial intelligence and immersive digital technologies in periodontal education: a systematic review
Source: Front Oral Health. 2026 Apr 9;7:1741033. doi: 10.3389/froh.2026.1741033 (PMC13103527; doi:10.3389/froh.2026.1741033)
Supplement: Supplementary file 2 [file Datasheet2.docx]

Supplementary Material

# Supplementary Tables

**Supplementary Table 1.** Search strategy

| Databases | Keywords |
| --- | --- |
| PubMed | ( "Artificial Intelligence"[Mesh] OR "Machine Learning"[Mesh] OR "Deep Learning"[Mesh] OR "Natural Language Processing"[Mesh] OR "Neural Networks, Computer"[Mesh] OR "Virtual Reality"[Mesh] OR "Augmented Reality"[Mesh] OR "Computer-Assisted Instruction"[Mesh] OR "Educational Technology"[Mesh] OR "artificial intelligence"[Title/Abstract] OR "AI"[Title/Abstract] OR "machine learning"[Title/Abstract] OR "deep learning"[Title/Abstract] OR "natural language processing"[Title/Abstract] OR "neural network*"[Title/Abstract] OR "virtual reality"[Title/Abstract] OR "augmented reality"[Title/Abstract] OR "mixed reality"[Title/Abstract] OR "immersive technology"[Title/Abstract] OR "intelligent tutoring system*"[Title/Abstract] OR "AI-based learning"[Title/Abstract] OR "computer-assisted instruction"[Title/Abstract] OR "chatbot*"[Title/Abstract] OR "educational technology"[Title/Abstract] ) AND ( "Education, Dental"[Mesh] OR "Students, Dental"[Mesh] OR "Periodontics/education"[Mesh] OR "dental education"[Title/Abstract] OR "dental curriculum"[Title/Abstract] OR "dental student*"[Title/Abstract] OR "periodontal education"[Title/Abstract] OR "periodontology education"[Title/Abstract] OR "teaching periodontics"[Title/Abstract] OR "periodontics training"[Title/Abstract] ) |
| Embase | ('artificial intelligence'/exp OR 'machine learning'/exp OR 'virtual reality'/exp OR 'augmented reality'/exp OR 'natural language processing'/exp OR 'computer assisted instruction'/exp OR 'artificial intelligence':ti,ab OR 'ai':ti,ab OR 'machine learning':ti,ab OR 'deep learning':ti,ab OR 'natural language processing':ti,ab OR 'neural network*':ti,ab OR 'virtual reality':ti,ab OR 'augmented reality':ti,ab OR 'mixed reality':ti,ab OR 'immersive technolog*':ti,ab OR 'intelligent tutoring system*':ti,ab OR 'ai-based learning':ti,ab OR 'chatbot*':ti,ab OR 'educational technology':ti,ab) AND ('dental education'/exp OR 'periodontics'/exp OR 'dental education':ti,ab OR 'dental curriculum':ti,ab OR 'dental student*':ti,ab OR 'periodontal education':ti,ab OR 'periodontology education':ti,ab OR 'teaching periodontics':ti,ab OR 'periodontics training':ti,ab) |
| Web of Science | TS= (“artificial intelligence” OR “AI” OR “machine learning” OR “deep learning” OR “natural language processing” OR “neural network” OR “virtual reality” OR “augmented reality” OR “mixed reality” OR “immersive technology” OR “intelligent tutoring system” OR “AI-based learning” OR “computer-assisted instruction” OR “chatbot” OR “educational technology”) AND (“dental education” OR “dental curriculum” OR “dental student” OR “periodontal education” OR “periodontology education” OR “teaching periodontics” OR “periodontics training”) |
| Cochrane Central Register of Controlled Trials | (“artificial intelligence” OR “AI” OR “machine learning” OR “deep learning” OR “natural language processing” OR “neural network” OR “virtual reality” OR “augmented reality” OR “mixed reality” OR “immersive technology” OR “intelligent tutoring system” OR “AI-based learning” OR “computer-assisted instruction” OR “chatbot” OR “educational technology”) AND (“dental education” OR “dental curriculum” OR “dental student” OR “periodontal education” OR “periodontology education” OR “teaching periodontics” OR “periodontics training”) |

**Supplementary Table 2.** List of excluded studies with reasons

| Study | Reason for exclusion |
| --- | --- |
| Almarghlani et al 2025 | No specific educational scenario described |
| Al-Saud et al 2017 | Not directly focus on periodontics |
| Bissell et al 2003 | Not involving artificial intelligence or immersive digital technologies |
| Danesh et al 2024 | No involvement of learners or educators in periodontal education context |
| Fanelli et al 2025 | No specific educational scenario described |
| Heym et al 2018 | Not involving artificial intelligence or immersive digital technologies |
| Parsegian et al 2024 | No specific educational scenario described |
| Qi et al 2013 | Not directly focus on periodontics |
| Zhang et al 2020 | Not directly focus on periodontics |

**Supplementary Table 3.** Technical specifications of artificial intelligence models and immersive digital platforms used in the included studies.

| **Study** | **Technology Category** | **Platform / Model** | **Version & Access** |
| --- | --- | --- | --- |
| Schittek Janda et al., 2004 [19] | Virtual Patient | custom-built web application (Programmed in PHP with a database backend). | Institutional prototype |
| Steinberg et al., 2007 [34] | Haptics-based virtual reality | PerioSim© (Hardware: PHANToM™; Software: modified GHOST™ API) | Institutional prototype |
| Luciano et al., 2009 [35] | Haptics-based virtual reality | Periodontal Simulator (Coin 3D library, GHOST toolkit, PHANToM) | Institutional prototype (Open-source graphics + Commercial haptics) |
| Wang et al., 2012 [36] | Haptics-based virtual reality | iDental (Hardware: Phantom Omni / Phantom Desktop; Software: GHOST SDK) | Institutional prototype |
| Yamaguchi et al., 2013 [20] | Haptics-based virtual reality | Custom Haptic VR Simulator (Modeled via FreeFormModeling) | Institutional prototype |
| Chehabeddine et al., 2021 [33] | Haptics-based virtual reality | Haptodont system (2x Geomagic Touch, Novint Falcon, Oculus Rift, Chai3D) | Institutional prototype (Open-source framework + Commercial hardware) |
| Zhang et al., 2021 [28] | Haptics-based virtual reality | UniDental system | Not given |
| Tak et al., 2023 [29] | 360° Virtual reality | 360° VR video (GoPro Fusion) viewed via YouTube & Google Cardboard HMD | Free / Consumer-grade accessible VR |
| Cheng et al., 2025 [21] | Haptics-based virtual reality | Oral digital virtual simulation training system (Beijing Zhonghui Virtual Reality Technology Research Institute) | Institutional prototype |
| Glick et al., 2022 [30] | Explainable artificial intelligence | U-Net Convolutional Neural Network | Not given |
| Boehm et al., 2025 [31] | Artificial intelligence-enhanced imaging | Overjet AI | Not given |
| Li et al., 2025 [37] | Large language models | ChatGPT | ChatGPT-3.5 (Free), ChatGPT-4 (Pro/Premium - August 3 Version) |
| Sabri et al., 2025 [38] (USA) | Large language models | ChatGPT, Google Gemini | ChatGPT-3.5 (Free), ChatGPT-4 (Premium), Google Gemini (Free) |
| Ramlogan et al., 2025 [39] | Large language models | ChatGPT-4, GPT-4o, CohereForAI, Claude, DeepSeek, Gemini, Mistral NeMo, Le Chat-Mistral, Llama-3.1, Llama- 3.3, Phi, Qwen | ChatGPT-4 (Sep 2023 & Mar 2024), GPT-4o (Omni, May 2024),CohereForAI (c4ai-command-r-plus), Claude (Claude3.5-sonnet), DeepSeek (DeepSeek-R1), Gemini (Gemini 2.0 flash), Mistral NeMo (Nemo-Instruct-2407), Le Chat-Mistral, Llama-3.1 (Nemotron-70B-Instruct), Llama-3.3 (70B-Instruct), Phi (phi-4 Q4_K_M), Qwen (Qwen2.5-72B) |
| Ma et al., 2025 [32] | Large language models | OpenAI GPT-4 | Not given |
